# The therapeutic potential of glucagon-like peptide-1 for persons with addictions based on findings from preclinical and clinical studies

**DOI:** 10.3389/fphar.2023.1063033

**Published:** 2023-03-30

**Authors:** Elisabet Jerlhag

**Affiliations:** Institute of Neuroscience and Physiology, Department of Pharmacology, The Sahlgrenska Academy at the University of Gothenburg, Gothenburg, Sweden

**Keywords:** addiction, dependence, reward, dopamine, gut–brain axis, appetite regulation

## Abstract

Although the multifaceted mechanisms underlying alcohol use disorder (AUD) have been partially defined, the neurobiological complexity of this disorder is yet to be unraveled. One of the systems that have gained attention in recent times is the gut–brain axis. Although numerous peptides participate in this axis, glucagon-like peptide-1 (GLP-1) plays a central role. GLP-1 is a crucial anorexigenic peptide, with potent abilities to reduce food intake and body weight. The physiological complexity of GLP-1 entails glucose homeostasis, gastrointestinal motility, and the release of insulin and glucagon. As reviewed in this study, acute or repeated treatment with GLP-1 receptor (GLP-1R) agonists decreases alcohol consumption in rodents. Moreover, the abilities of alcohol to promote hyperlocomotion, dopamine release in the nucleus accumbens, and reward in the conditioned place preference paradigm are all suppressed by GLP-1R ligands. Moreover, activation of GLP-1R suppresses the motivation to consume alcohol, alcohol-seeking behaviors, and relapse drinking in male rodents. Similarly, abstinence symptoms experienced during alcohol withdrawal are attenuated by activation of the GLP-1 pathway. On a similar note, the activation of GLP-1 receptors within areas of the brain that are processing reward modulates these alcohol-related responses. Another area that is crucial for this ability is the nucleus of the solitary tract, which is where GLP-1 is produced and from which GLP-1-containing neurons project to areas of reward. These findings may have clinical relevance as AUD is associated with polymorphisms in GLP-1-related genes. Although a GLP-1R agonist does not alter alcohol intake in AUD patients, it reduces this consumption in a sub-population of obese AUD individuals. Given the uncertainty of this outcome, additional clinical studies of obese AUD patients should explore the effects of the GLP-1R agonists on alcohol intake and body weight. Furthermore, GLP-1 receptors modulate the behavioral and neurochemical responses to addictive drugs. Taken together, these preclinical and clinical findings imply that the GLP-1 pathway plays a role in the complex mechanisms regulating alcohol and drug consumption patterns, unveiling a novel aspect of addiction medicine.

## 1 Introduction

The gut–brain peptide, glucagon-like peptide-1 (GLP-1), is encoded by the proglucagon (PPG) gene, which is expressed in the intestines and pancreas [for a review, see Holst (2007)]. In the intestine and pancreas, GLP-1 is produced and secreted upon meal ingestion, and after its release, it acts as an incretin and is involved in satiation signaling (Holst, 2007). In the intestine, GLP-1 production colocalizes with the production of glucose-dependent insulinotropic polypeptide (GIP) or peptide YY (PYY), and they share physiological properties, such as glucose control (Holst, 2007). Moreover, GLP-1 production has been shown in some neurons of the nucleus of the solitary tract (NTS) in the brain stem [for a review, see Holst (2007)]. The projections of the PPG-containing neurons of the NTS are widespread. In particular, they target areas that are important for homeostatic feeding, such as the hypothalamus, and for hedonic responses, such as the nucleus accumbens (NAc), ventral tegmental area (VTA), and laterodorsal tegmental area (LDTg) (Alhadeff et al., 2012; Graham et al., 2020). Although less extensively studied and characterized, the *PPG* gene is also expressed in the olfactory bulb (Merchenthaler et al., 1999), amygdala, striatum, and hippocampus (Vallof et al., 2019a). It is also most likely that the GLP-1 produced in the body is different from that produced in the brain.

The extensive physiological and behavioral effects of GLP-1 are mediated by the GLP-1 receptors (GLP-1R), which are expressed in both the central and autonomic nervous systems (Merchenthaler et al., 1999; Graham et al., 2020). One of the best-known activities of GLP-1 is the normalization of plasma glucose levels, achieved through the facilitation of insulin secretion and lowering of glucagon secretion (Holst and Seino, 2009). These incretin functions have led to the approval of GLP-1R agonists for the treatment of type II diabetes (Kreymann et al., 1987). Another prominent feature of GLP-1 is its anorexigenic properties. Indeed, activation of GLP-1R reduces both homeostatic and hedonic feeding, and it decreases appetite (Tang-Christensen et al., 1996; Turton et al., 1996; Langhans, 2000; Naslund et al., 2005; Hayes et al., 2008; Alhadeff et al., 2012; Mietlicki-Baase et al., 2013; Wang et al., 2015). Considering that some of the GLP-1R agonists also decrease body weight, these have received approval for the treatment of obesity in humans [for a review, see Srivastava and Apovian (2018)]. In addition, GLP-1 affects gastric emptying, cardiac functions, and brain-related behaviors, such as reward, emotion, motivation, and learning (Holst, 2007).

Following ingestion of a meal, the GLP-1 levels rapidly increase. However, the peptide is degraded by the enzymes DPP-IV and neutral endopeptidase 24.11 (Holst, 2007). Therefore, limited amounts of GLP-1 reach the brain after its release from the intestine. This rapid degradation of GLP-1 has motivated the development of long-acting GLP-1R agonists, which are protected from DPP-IV degradation and can be used clinically (Kalra et al., 2021). These pharmaceuticals include exendin-4 (Ex4) and liraglutide, which are injected subcutaneously twice daily. Dulaglutide and semaglutide are two additional pharmaceutical agents that are administered subcutaneously weekly once, with the latter also being available as a peroral treatment. Other GLP-1 based drugs, such as the DPP-IV inhibitors sitagliptin and linagliptin, are also available for the treatment of type II diabetes. Although the GLP-1R antagonist exendin-9-39 (Ex9) is not used clinically, it has been used for research purposes because it blocks the endogenously released GLP-1.

GLP-1 is a multifaceted peptide that can modulate alcohol-related responses. This activity of GLP-1 has been demonstrated in both preclinical and clinical studies, and the results support the hypothesis that the GLP-1 pathway regulates the processes of alcohol use disorder (AUD). AUD is a chronic relapsing disorder with a complex neurobiological pathophysiology. As extensively described elsewhere [for a review, see Koob (2014)], AUD involves recurring cycles of specific behaviors, involving an initial binge/reward phase followed by compulsive alcohol intake, which transitions into drinking with an aim to reduce the negative effects and the presence of withdrawal symptoms. During this latter stage, craving for alcohol and the subsequent relapse toward drinking are typical behaviors for individuals with AUD. These cycles are modulated by several neurocircuits, whereby the mesolimbic dopamine pathway appears to be important for binge drinking, compulsive alcohol drinking, and craving for alcohol (Jayaram‐Lindström et al., 2016). Moreover, the dopamine pathway mediates the rewarding aspects of alcohol, given that alcohol releases dopamine in humans and rodents, an effect that is associated with the rewarding experience of drinking for humans (Jayaram‐Lindström et al., 2016). The mesolimbic dopamine system consists of dopamine projections from the VTA to target areas such as the NAc shell or amygdala. However, it should be noted that other neurocircuits are also important for AUD processes. Although extensive research has identified numerous neurobiological underpinnings of AUD, more recent advances in animals and humans reveal that the GLP-1 pathway plays a role. This review summarizes the available preclinical and clinical literature regarding how the GLP-1 pathway modulates alcohol-mediated responses.

Although a systematic review selects articles by PRISM, JBI, PICOS, or Cochrane, the present review uses keywords and free-text words in multiple databases. Moreover, the design/results of the selected articles were briefly checked for quality, and the review should be considered as a narrative literature review. The selected design may be considered a limitation as a systematic review provides the highest level of reliability.

## 2 Interactions between the GLP-1 pathway and alcohol

**FIGURE 1:**
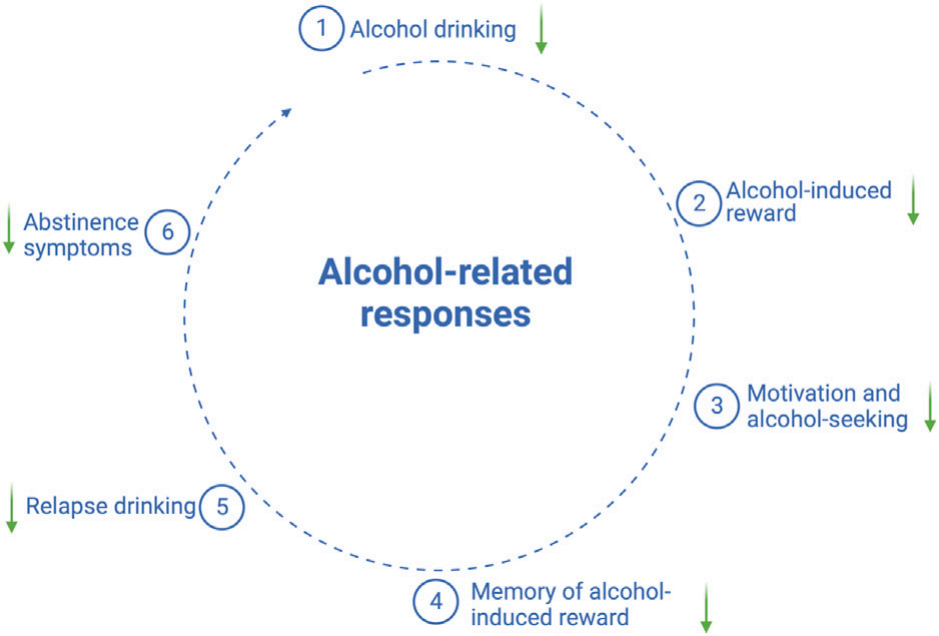
Schematic illustration of alcohol-related responses that are reduced (↓) by GLP-1 receptor agonists; the illustration is created using bioreder.com.

As shown in Figure 1, the accumulated preclinical findings imply that the GLP-1 pathway modulates alcohol-mediated responses, which include the following: 1) alcohol drinking, 2) alcohol-induced behaviors and neurochemistry, 3) the motivation to consume alcohol and alcohol-seeking, and 4) relapse drinking. Moreover, the ability of GLP-1 signaling to interact with alcohol-related effects also appears to hold true in the clinical situation, the findings for which are reviewed herein.

### 2.1 The GLP-1 pathway influences different alcohol-mediated responses in animals

#### 2.1.1 Alcohol consumption

Using two different bottle choice models, alcohol drinking over a prolonged period of time can be monitored in animals (Sanchis-Segura and Spanagel, 2006). Although the rats may not be addicted to the alcohol, the intake levels reflect different aspects of hazardous consumption of alcohol. The first publication that reported an association between GLP-1R and alcohol intake showed that acute administration of Ex4 reduced both alcohol drinking and preference for alcohol in male rats that were exposed to alcohol for 10 weeks before treatment (Egecioglu et al., 2013a). Since then, additional studies of alcohol-dependent male rats have confirmed this reduction in alcohol drinking following acute Ex4, GLP-1, or liraglutide injection (Davis et al., 2012; Shirazi et al., 2013; Vallof et al., 2015; Marty et al., 2020). On a similar note, Ex4 or liraglutide lowers the intake of alcohol in non-human primates (Thomsen et al., 2019). Although it has not been compared in any other studies, the efficacy with which liraglutide lowers alcohol consumption is greater than the corresponding efficacy of Ex4 in non-human primates (Thomsen et al., 2019), indicating differences between these GLP-1R agonists. The finding that acute injection of semaglutide reduces alcohol intake in male rats (Marty et al., 2020) provides further evidence of the ability of this GLP-1R agonist to suppress alcohol consumption.

Similar to the results obtained with acute injection, repeated liraglutide treatment (six injections over 2 weeks) of alcohol-experienced male rats has been shown to decrease both alcohol consumption and preference for alcohol (Vallof et al., 2015). Long-acting GLP-1R analogs, such as dulaglutide, have the advantage of weekly rather than daily injections. Indeed, once-weekly injections (for either 5 weeks or 9 weeks) of dulaglutide have been shown to reduce both alcohol consumption and preference for alcohol in male rats throughout the treatment period (Vallof et al., 2020). In alcohol tests in which Ex4, liraglutide, or dulaglutide was injected repeatedly, there was no development of tolerance toward the GLP-1R agonists tested (Suchankova et al., 2015; Vallof et al., 2015; Vallof et al., 2020). Moreover, the decline in alcohol drinking persisted after treatment termination (Vallof et al., 2020). These dulaglutide-mediated reductions in alcohol drinking and preference for alcohol were also evident in female rats (Vallof et al., 2020), an outcome that was not seen previously. However, it should be noted that the reductions were more pronounced in male rats than in female rats (Vallof et al., 2020), suggesting that sex differences in treatment outcome should be further evaluated in upcoming studies. Although the studies described here provide evidence of a profound reduction in alcohol drinking after GLP-1R agonists, these findings could be extended in an additional study showing that Ex4 alters the pattern of alcohol drinking. Indeed, it has been demonstrated that in group-housed male mice, Ex4 increases the latency to the first alcohol bout and decreases the number of alcohol drinks consumed (Thomsen et al., 2017). As the presented experiments do not report absolute changes in alcohol drinking after GLP-1R agonist treatment, the effectiveness of the different agonists cannot be compared. This can be considered a limitation and should be addressed in future studies.

Although the aforementioned preclinical studies with GLP-1R reveal a role for the receptor in alcohol drinking, the importance of GLP-1 itself has not been studied as intensely. GLP-1 is mainly found in the circulation, where it is rapidly degraded after secretion (Holst, 2007), and the relative importance of central *versus* peripheral GLP-1 for alcohol drinking is uncertain. In this context, infusion of the GLP-1R antagonist into the NTS prevents the suppressive effect of Ex4 on alcohol-induced behaviors (Vallof et al., 2019b). Moreover, attenuation of endogenous GLP-1 in the VTA in the brain (Shirazi et al., 2013) increases alcohol intake in male rats. In contrast, the alcohol intake remains unaltered after pharmacologic manipulations that increase the level of circulating GLP-1 (Marty et al., 2020). In particular, the alcohol intake is unaffected after treatment with either an agonist directed toward protein receptor 119 in the L cells that synthesize GLP-1 or the injection of a DPP-IV inhibitor (Marty et al., 2020). Collectively, these studies suggest that central rather than peripheral GLP-1/GLP-1R mediates the alcohol responses. Further studies exploring these aspects are needed.

#### 2.1.2 Alcohol-induced reward

Although high-level consumption of alcohol over a prolonged period of time is important for the development of AUD, other fundamental aspects of this complex disorder need to be considered. The initial phase is, to a large extent, driven by reward (Koob, 2014), which is regulated by the GLP-1 pathway. In animal models, this alcohol-induced reward is characterized by activation of the mesolimbic dopamine system, and it can be studied in preclinical models that involve hyperlocomotion, dopamine release in the NAc, and reward in the conditioned place preference (CPP) paradigm (Sanchis-Segura and Spanagel, 2006). On this note, the alcohol-induced hyperactivity, dopamine release in the NAc, and its reward measured using the CPP paradigm are all blocked by Ex4 in male mice (Egecioglu et al., 2013a; Shirazi et al., 2013). In addition, liraglutide inhibits the ability of alcohol to cause locomotor stimulation or to release dopamine in the NAc in male mice (Vallof et al., 2015). Apart from reward, this initial AUD phase is influenced by the motivation to consume alcohol, which is another factor controlled by GLP-1R in male rats. In rodents, this behavior can be studied in the operant self-administration model (Sanchis-Segura and Spanagel, 2006). Specifically, acute administration of Ex4 or repeated liraglutide treatment decreases, in a dose-dependent manner, the motivation to obtain alcohol in the operant self-administration paradigm (Egecioglu et al., 2013a; Vallof et al., 2015; Bornebusch et al., 2019). As further discovered using this model, Ex4 prevents alcohol-seeking in the progressive ratio test (Egecioglu et al., 2013a). Another feature of the transition from regular alcohol drinking into AUD is the memory consolidation of alcohol reward, which can be studied in the CPP paradigm (mCPP) (Sanchis-Segura and Spanagel, 2006). In male mice, this alcohol-induced mCPP is blocked by the GLP-1R agonists Ex4 and GLP-1 (Egecioglu et al., 2013a; Shirazi et al., 2013), whereas liraglutide does not have this ability (Vallof et al., 2015). This finding may relate to the different abilities of these agonists to target brain regions that are crucial for memory processes (Salinas et al., 2018). Later on, in the addiction cycle, relapse drinking after alcohol withdrawal is an important aspect of AUD, and this can be modeled using the alcohol deprivation test in rats (Sanchis-Segura and Spanagel, 2006). Indeed, this relapse drinking is prevented by Ex4 in both single-housed (Vallof et al., 2015) and group-housed (Thomsen et al., 2017) male rodents. Furthermore, escalated relapse drinking after repeated withdrawal phases is attenuated by another GLP-1R agonist, AC3174 (Suchankova et al., 2015). Thereafter, the addiction cycle transitions into drinking to control the negative effects and abstinence symptoms; these are additional aspects controlled by GLP-1R and/or GLP-1. Indeed, liraglutide or sitagliptin (a DPP-IV inhibitor) can reduce the withdrawal symptoms during alcohol abstinence (Sharma et al., 2014a; Sharma et al., 2014b). In contrast to other alcohol responses, the withdrawal symptoms linked to alcohol appear to involve circulating GLP-1, which suggests that differentiated GLP-1 pathways are important for the different aspects of the AUD cycle.

### 2.2 Several GLP-1R-containing brain regions regulate alcohol-mediated responses in rodents

**FIGURE 2:**
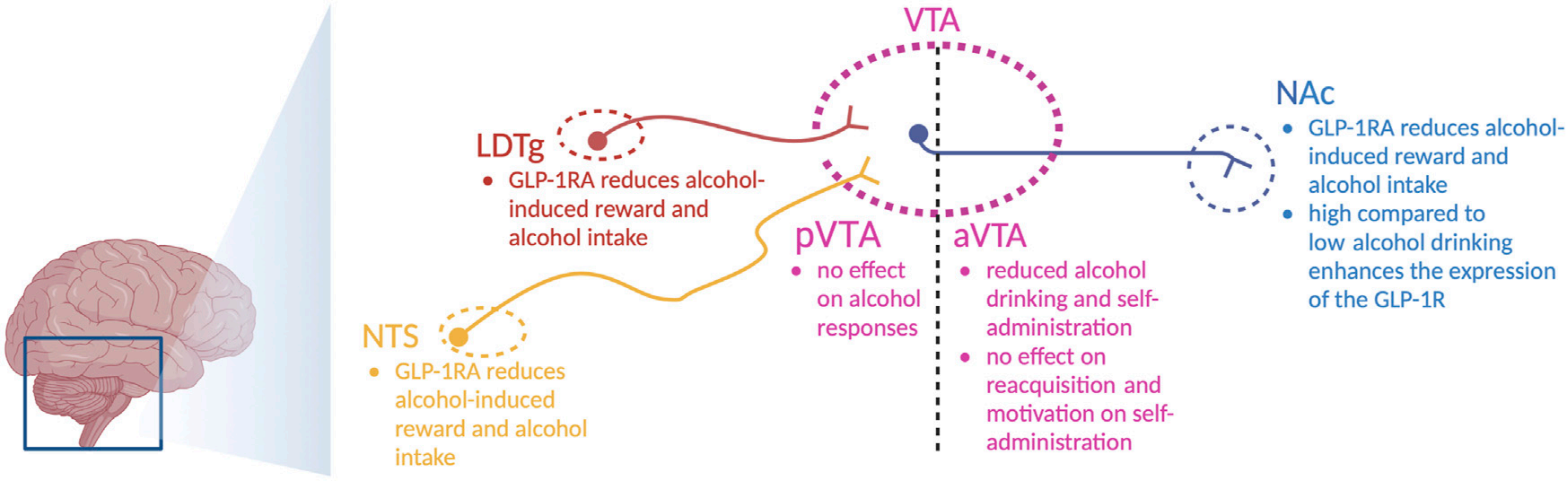
Schematic illustration of selected areas in which GLP-1 receptor agonists act to regulate alcohol-related responses in rodents. Indicated are the nucleus of the solitary tract (NTS), nucleus accumbens (NAc), ventral tegmental area (VTA), anterior VTA (aVTA), posterior VTA (pVTA), laterodorsal tegmental area (LDTg), and GLP-1 receptor agonists (GLP-1RA); the illustration is created using bioreder.com.

After systemic injections of the aforementioned GLP-1R agonists, the roles of central and peripheral receptors cannot be distinguished. This distinction was explored in a study where the Ex4-induced decline in alcohol drinking was blunted in central GLP-1R knockout mice, whereas the effect of Ex4 remained intact after peripheral GLP-1R knockout (Sirohi et al., 2016). Although these studies point toward the importance of central receptors, the brain nodes and circuits cannot be determined. One circuit of interest is the GLP-1-producing neurons from the NTS that target the LDTg, VTA, and NAc (Figure 2) (Rinaman, 2010). These regions mediate reward, motivation, and AUD processes, and they are important for other gut–brain axis peptides to regulate alcohol-related responses in rodents [for a review, see Shevchouk et al. (2021)].

On this note, Ex4 introduction into the NTS lowers alcohol drinking in male rats (Vallof et al., 2019b). Similarly, the abilities of alcohol to cause hyperlocomotion, dopamine release in the NAc, and mCPP are suppressed after local infusion of Ex4 into the NTS (Vallof et al., 2019b). Moreover, a reduction in alcohol drinking is evident after Ex4 is infused into the NTS (Vallof et al., 2019b). Inhibition of GLP-1R in the NTS (by Ex9) prevents Ex4 from executing its attenuation of alcohol-induced hyperlocomotion (Vallof et al., 2019b), which indicates that circulating GLP-1 may act *via* NTS to regulate alcohol responses. When it comes to the areas targeted by the NTS, the effects of Ex4 infusions on alcohol-mediated behaviors and neurochemistry diverge. Indeed, local Ex4 infusion into the LDTg suppresses alcohol-induced locomotor stimulation, dopamine release in the NAc, memory of alcohol reward, and alcohol drinking in male rodents (Vallof et al., 2019a). When Ex4 is infused into the anterior part of the VTA, it does not alter alcohol-mediated behaviors (Vallof et al., 2019a). In contrast, Ex4 infusion into the posterior part of the VTA reduces alcohol drinking (Shirazi et al., 2013; Colvin et al., 2020) and decreases alcohol self-administration, without affecting the extinction-induced reacquisition of alcohol self-administration or the motivation to consume alcohol (Dixon et al., 2020). The finding that Ex4 infusion into the anterior or posterior VTA alters the alcohol response in different ways is not surprising, as this is a heterogeneous area of the brain. Dopamine neurons of the VTA target the NAc, as evidenced by the appearance of fluorescently labeled Ex4 (Fluro-Ex4) in the NAc following systemic administration (Hernandez et al., 2019). Specifically, Fluor-Ex4 binds to the GLP-1R on the neurons and on the astrocytes within this area (Hernandez et al., 2019). After Ex4 infusion into the NAc, the abilities of alcohol to cause locomotor stimulation and mCPP are attenuated in male mice (Vallof et al., 2019a). Likewise, this treatment reduces alcohol drinking in both male and female rats (Abtahi et al., 2018; Vallof et al., 2019a; Colvin et al., 2020). An interaction between alcohol and GLP-1R in the NAc is further supported as the expression of the *GLP-1R* gene is elevated in high-level alcohol-drinking male rats compared to low-level alcohol-drinking male rats (Vallof et al., 2019a).

Other GLP-1R-containing brain areas that participate in this suppression of alcohol-linked behaviors are the lateral hypothalamus and dorsomedial hippocampus (Colvin et al., 2020). One human study has contributed to this identification as it shows that the hippocampal expression of GLP-1R is higher in patients with AUD than in controls (Farokhnia et al., 2022). Within the brain, GLP-1R is widely expressed, and the roles of areas with a high density of GLP-1R, e.g., the lateral septum, remain to be elucidated. Moreover, the key circuits and neurotransmitters should be explored, whereby the GABAergic system is a candidate that deserves attention, given that GLP-1R is located presynaptically on such nerve terminals (Rebosio et al., 2018).

### 2.3 GLP-1R activation reduces alcohol drinking in overweight patients with AUD

Although numerous preclinical studies have revealed that activation of the GLP-1 pathway suppresses alcohol-related responses, only a handful of clinical studies have explored this interaction in humans. The first indication was provided by a small, pilot study in patients with diabetes. Thus, patients treated with liraglutide reported reduced alcohol intake compared to patients who received other diabetic treatments (Kalra, 2011). This tentative interaction was explored in a recently conducted randomized clinical trial of patients with AUD treated with the GLP-1R agonist exenatide (similar to Ex4). In this study, exenatide does not alter alcohol intake in AUD patients who were of regular weight (Klausen et al., 2022). However, in this initial report, exenatide modestly reduced alcohol intake in AUD patients who were overweight (Klausen et al., 2022). Given the uncertainty of this initial clinical trial, additional studies exploring the effect of different GLP-1R agonists on alcohol drinking in overweight AUD patients are needed in the future. In addition, AUD and high alcohol intake are associated with polymorphisms in the *GLP-1R* gene (Suchankova et al., 2015). Moreover, these *GLP-1R* polymorphisms are associated with higher breath concentrations of alcohol and increased intravenous infusion of alcohol in social drinkers (Suchankova et al., 2015). However, this association was not found in another human genetic study (Tsermpini et al., 2022). Furthermore, *GLP-1R* expression in the hippocampus is higher in AUD patients compared to controls (Farokhnia et al., 2022). Although these studies indicate that GLP-1R influences alcohol intake, the converse has also been demonstrated, in that alcohol intake consistently reduces the plasma levels of GLP-1 in humans (Farokhnia et al., 2022).

## 3 Preclinical studies reveal interactions between GLP-1R and addictive drugs

**FIGURE 3:**
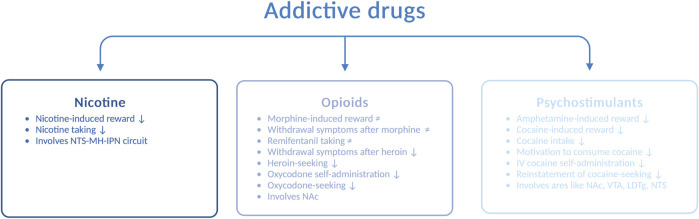
Schematic illustration of various drug-related responses that are reduced (↓) or unaltered (≠) by GLP-1 receptor agonists. Abbreviated are the nucleus of the solitary tract (NTS), medial habenula (MH), interpeduncular nucleus (IPN), nucleus accumbens (NAc), ventral tegmental area (VTA), laterodorsal tegmental area (LDTg), and intravenous (IV); the illustration is created using bioreder.com.

In addition to alcohol, the increased use of and dependence upon addictive drugs are major concerns in today’s society. Interactions between the GLP-1 pathway and addictive drugs, such as nicotine, opioids, and psychostimulants, have been demonstrated in rodents (Figure 3), and these are reviewed in the following sections.

### 3.1 Nicotine

AUD is often accompanied by nicotine dependence, as nicotine enhances the urge to consume alcohol and the two drugs enhance their corresponding behavioral responses [for a review, see Larsson and Engel (2004)]. The notion that GLP-1R influences the response to nicotine falls may indicate that the two drugs have similar neurobiological substrates. Specifically, Ex4 blocks the ability of nicotine to activate the mesolimbic dopamine system and cause reward (Egecioglu et al., 2013b), and it also suppresses nicotine intake (Tuesta et al., 2017) in male mice. Furthermore, the self-administration of nicotine is enhanced after knockout of the *GLP-1R* gene (Tuesta et al., 2017). Although numerous brain areas may be involved in the modulation of this interaction, an initial study has shown that the circuit between the NTS and the medial habenula (i.e., the interpeduncular nucleus) participate in the nicotine-GLP-1R cross-talk (Tuesta et al., 2017). In addition to GLP-1R, the peptide itself appears to be important for this interaction, as the increased levels of GLP-1 after DPP-IV inhibition (with sitagliptin) reduce nicotine consumption in male mice (Tuesta et al., 2017).

### 3.2 Opioids

Although opioids such as morphine, remifentanil, heroin, and oxycodone largely share a common pharmacologic mechanism of action, namely, acting on opioid receptors, the ability of GLP-1R to regulate the behavioral responses to these opioids varies. Ex4 does not alter the ability of morphine to cause hyperactivity, CPP, and withdrawal symptoms or remifentanil intake in male rodents (Bornebusch et al., 2019). In contrast to its receptor, circulating GLP-1 is engaged for morphine-mediated behaviors. Indeed, DPP-IV inhibition suppresses the expression, acquisition, and reinstatement of morphine-induced CPP (Lupina et al., 2020). When considering heroin, Ex4 attenuates withdrawal symptoms during heroin abstinence and diminishes heroin-seeking in male rats (Douton et al., 2021). Similarly, Ex4 reduces oxycodone self-administration and oxycodone-seeking (Zhang et al., 2020). This effect is also observed after specific activation of GLP-1R in the NAc and most likely those receptors located on medium spiny neurons that express the dopamine-D1 receptors or dopamine-D2 receptors (Zhang et al., 2020). Given the divergent outcomes of these studies, the efficacies of GLP-1R agonists for opioid-related responses need to be investigated in greater detail.

### 3.3 Psychostimulants

In addition, the behavioral and neurochemical responses to psychostimulants such as amphetamine and cocaine are modulated by GLP-1R. The hyperactivity (Erreger et al., 2012; Egecioglu et al., 2013c), reward in the CPP test, and dopamine release in the NAc (Egecioglu et al., 2013c) observed after amphetamine intake are reduced by acute systemic administration of Ex4 to male mice. Furthermore, this suppression involves central rather than peripheral GLP-1R (Sirohi et al., 2016). Although an increased circulating level of GLP-1 does not modulate alcohol-related responses, it mediates the behavioral responses to amphetamine (Lautar et al., 2005). The abilities of cocaine to cause hyperlocomotion (Egecioglu et al., 2013c; Sorensen et al., 2015) and reward in the CPP test (Egecioglu et al., 2013c; Graham et al., 2013; Sorensen et al., 2015) are attenuated by Ex4. Moreover, cocaine-related behaviors are enhanced in *GLP-1R* knockout mice (Harasta et al., 2015), providing further evidence for the GLP-1R–cocaine interaction. In addition to affecting the behavioral responses to cocaine, Ex4 reduces its neurochemical effects. Indeed, Ex4 suppresses the cocaine-induced dopamine overflow in the NAc (Egecioglu et al., 2013c; Sorensen et al., 2015) and phasic dopamine release in the NAc core (Fortin and Roitman, 2017). On a similar note, GLP-1R modulates the consumption of cocaine and the motivation to consume cocaine later in the addiction cycle. Thus, in male rats, intravenous cocaine self-administration (Sorensen et al., 2015; Fortin and Roitman, 2017; Hernandez et al., 2019) and the reinstatement of cocaine-seeking (Hernandez et al., 2018) are decreased after Ex4 injection.

Similar to the alcohol-related responses, GLP-1R within the NTS, LDTg, VTA, and NAc modulates the behavioral responses induced by cocaine. Initial findings from an investigation of this circuit reveal that either Ex4 infusion (Schmidt et al., 2016) or expression of *PPG* mRNA (Hernandez et al., 2018) in the NTS decreases cocaine self-administration in male rats. This underlines the importance of both the GLP-1R and GLP-1 itself for cocaine intake. When considering LDTg, Ex4 infusion into this area suppresses cocaine-related behaviors (Hernandez et al., 2020). For the VTA, genetic suppression of GLP-1R augments cocaine seeking (Schmidt et al., 2016), whereas pharmacological activation amplifies it (Schmidt et al., 2016; Hernandez et al., 2018). The importance of the aforementioned circuit is further supported by the fact that Ex4 infusion into the NAc, an area targeted by dopamine neurons of the VTA, attenuates cocaine seeking (Hernandez et al., 2019). Moreover, Ex4 prevents the cocaine-induced activation of neurons of the NAc (Sorensen et al., 2015) and increases the intrinsic excitability of the medium spiny neurons in cocaine-exposed rats (Hernandez et al., 2019).

Another area relevant to the GLP-1–cocaine link is the lateral septum, which exhibits the highest level of expression of GLP-1R in the brain and modulates the activity within the mesolimbic system. In line with this notion, pharmacologic or genetic suppression of GLP-1R in the lateral septum attenuates the ability of cocaine to activate the mesolimbic dopamine system, as assessed by hyperactivity, reward in the CPP test, and dopamine enhancement in the NAc (Harasta et al., 2015; Reddy et al., 2016). These effects are tentatively mediated *via* arachidonic acid and the increased expression of the dopamine transporter within the lateral septum (Reddy et al., 2016).

Although the vast majority of studies have revealed interactions between cocaine responses and GLP-1R, some studies have reported correlations between the responses to cocaine and the plasma levels of GLP-1. In contrast to alcohol, cocaine intake increases the GLP-1 levels in the plasma in rodents (You et al., 2019) and reduces the corresponding levels in humans (Bouhlal et al., 2017). Further evidence for the involvement of circulating GLP-1 in the responses to cocaine is that in humans, higher plasma levels of GLP-1 are associated with the subjective experience of cocaine (Bouhlal et al., 2017).

## 4 Confounding factors

Although the aforementioned studies show that GLP-1R agonists and/or GLP-1 modulate the responses to alcohol and addictive drugs, various limitations and confounding factors influence the conclusions that can be drawn from the acquired data.

A potential confounding factor is a general effect on ingestive behaviors, given that the GLP-1 pathway reduces the levels of the intake of alcohol, addictive drugs, and food. However, this is unlikely, as Ex4 reduces intravenous self-administration of alcohol in male mice (Sorensen et al., 2016) and the GLP-1R agonist does not reduce water intake. Moreover, reductions in alcohol/drug responses (hyperactivity, dopamine release, and CPP), which are not influenced by ingestion, are observed. Although each of the different GLP-1R agonists tested reduced alcohol drinking, the receptor selectivity profiles of these agonists should be addressed as a confounding factor. Nonetheless, it appears less likely that other receptors modulate the observed effects, as a GLP-1R antagonist (Ex9) blocks the ability of the GLP-1R agonists to exhibit their effects on alcohol drinking (Marty et al., 2020). Although calories and augmented alcohol metabolism are other aspects that might influence the data, these seem unlikely because the GLP-1 pathway suppresses drug responses that are independent of calories and because liraglutide does not change the blood alcohol levels (Vallof et al., 2015). The most plausible confounding factor is malaise, which is a common side effect of the GLP-1R agonists used (Kanoski et al., 2012). Although indirectly excluding this factor, the tested GLP-1R agonists act as follows: 1) they do not cause a CPP *per se* as an indication of conditioned aversion (Cagniard and Murphy, 2012), 2) they are used in dosages that are not associated with malaise (Cagniard and Murphy, 2012; Kanoski et al., 2012; Mietlicki-Baase et al., 2013; Baisley and Baldo, 2014; Mietlicki-Baase et al., 2017; Rodriguez et al., 2018), 3) they enhance water intake, and 4) they reduce alcohol/drug responses that are unaffected by aversion [for a review, see Shevchouk et al. (2021)]. On a final note, preclinical models are combined to reflect the responses in animals, although animals cannot be diagnosed with an addiction, so this is a limitation associated with preclinical studies.

When tentatively treating individuals with alcohol use disorder using GLP-1R agonists, it is important to take into account the potential aversive effects. One of the most common side effects reported for all GLP-1R agonists is malaise (Kanoski et al., 2012). In addition, GLP-1R agonists have been linked to an increased risk of pancreatitis, which could be problematic for AUD patients, as some of them may already have pancreatitis. As a result, it is recommended to monitor pancreatic function when treating AUD patients or to avoid GLP-1R agonists altogether in those with pancreatitis. Furthermore, it is important to consider the potential for weight loss associated with some GLP-1R agonists, which may not be beneficial for AUD patients with a low body weight. Additionally, lean individuals have reported more side effects compared to overweight individuals when treated with GLP-1R agonists. Therefore, primarily overweight individuals with AUD should be considered for treatment with GLP-1R agonists.

## 5 Future directions and summary

As summarized previously, GLP-1R, and to some extent the peptide itself, influences the behavioral and neurochemical outcomes of alcohol and addictive drugs, such as nicotine, opioids, amphetamine, and cocaine. To follow up on these initial studies, the distinct function of GLP-1 compared to its receptor and the roles of central *versus* peripheral pathways in drug/alcohol responses should be determined. Likewise, the roles of GLP-1 in areas such as the olfactory bulb (Merchenthaler et al., 1999), amygdala, striatum, and hippocampus (Vallof et al., 2019a) in terms of addiction are unknown and should be investigated.

The processes of passage through the blood–brain barrier and penetration into deeper brain regions differ between the GLP-1R agonists (Salinas et al., 2018). Moreover, the different agonists act differently on the second messenger systems. How this aspect influences the treatment outcome with respect to alcohol/drug-related responses is a tentative focus of future studies. It should also be taken into consideration that the treatment outcome after the GLP-1R agonists may be different for rats drinking alcohol or not, as alcohol consumption enhances traversing of the blood–brain barrier (Rubio-Araiz et al., 2017). Different treatment outcomes linked to the different GLP-1R agonists should also be evaluated, since dulaglutide (in contrast to Ex4 or liraglutide) augments the learning of skilled reach foraging (Vestlund et al., 2020). Semaglutide, which is the most recently approved GLP-1R agonist, is of great interest because it can be administered orally weekly once. Although a high dose of semaglutide reduces alcohol intake in male rats (Marty et al., 2020), additional preclinical studies are needed to explore the effects of semaglutide on alcohol drinking in various preclinical models of AUD in rats of both sexes.

As the addiction cycle involves the repetition of different phases and the diagnosis is defined by different symptoms, the influence of the GLP-1 pathway on each of these should be examined in detail. Addictions are complex disorders, and a combination of preclinical models is used to gain insights into the neurobiological underpinnings. Although the results obtained from these preclinical models translate to humans, different mechanisms exist in animals and humans, the investigation of which is a topic for future studies. As concomitant abuse of different drugs is common in today’s society, the effects of GLP-1R agonists on the intake on multiple addictive drugs in a situation where the rats can choose between different drugs should be tested.

According to the articles summarized previously, the NTS and its projections to the LDTg, VTA, and NAc participate in the interactions between the GLP-1 pathway and alcohol/addictive drugs. However, since the expression of GLP-1R is widespread, additional areas of the brain are most likely to participate in these interactions. An additional priority of upcoming studies will be to define the key neurocircuits. To date, GLP-1R has been identified presynaptically on GABAergic nerve terminals (Rebosio et al., 2018) and on medium spiny neurons that express the dopamine-D1 or dopamine-D2 receptors (Zhang et al., 2020). Moreover, dulaglutide reduces *ex vivo* dopaminergic neurotransmission in the amygdala and striatum (Vallof et al., 2020). Therefore, initial studies could be designed to explore whether and how GLP-1R agonists alter GABAergic and dopaminergic pathways in the brain. In support of this idea, the gut–brain axis peptide ghrelin enhances dopamine release in the VTA, an effect that modulates the ability of alcohol to activate the mesolimbic dopamine system (Edvardsson et al., 2021). Although the aforementioned studies indicate that central mechanisms modulate the ability of GLP-1R agonists to reduce alcohol intake, other mechanisms such as the gut microbiota may participate. Indeed, alcohol and GLP-1R agonists modify the gut microbiota composition [for reviews, see Bajaj (2019); Sayehmiri et al. (2022)]. However, if the interaction between microbiota, GLP-1, and alcohol is dependent or separate remains to be determined.

Another aspect that should be explored is the treatment effect in female rodents. Indeed, dulaglutide shows sex-specific treatment differences, as males reduce their intake levels more than females, and the treatment effect persists after discontinuation in males but not in females (Vallof et al., 2020). Since dulaglutide decreases monoaminergic neurotransmission in the amygdala in males but in the striatum in females (Vallof et al., 2020), the possibility that different mechanisms of action contribute to the decline in alcohol drinking in males and females should be considered.

Although these initial studies directly manipulate GLP-1R or GLP-1, other ways to influence the GLP-1 pathway are less studied. For instance, ketogenic diets or ketone supplements alter the production of GLP-1 (Wallenius et al., 2020). Thus, the possibility that ketogenic diets or ketone supplements alter different phases of the addiction cycle should be explored in detail. In support of this idea, initial human and rodent studies have revealed that ketogenic diets reduce the withdrawal symptoms after alcohol exposure (Derr et al., 1983; Wiers et al., 2021) and that a ketogenic diet reduces alcohol intake in male rats (Wiers et al., 2021).

GLP-1 is produced together with anorexigenic peptide GIP, and the combination of the GLP-1R agonist and GIP synergistically reduces the level of feeding and body weight. Another treatment combination with synergistic effects on homeostatic parameters involves GLP-1R and the amylin receptor agonist. As AUD is a heterogeneous disorder with a multifaceted pathophysiology, the possibility that combinations of GLP-1 and other gut–brain axis peptides synergistically reduce alcohol drinking should be explored in future studies.

From the clinical perspective, *GLP-1R* gene polymorphism is associated with AUD, and exenatide moderately reduces alcohol drinking in overweight AUD patients. Additional clinical studies are needed to explore this research field in more detail. One such study should explore the treatment outcomes of GLP-1R agonists on the subjective experiences associated with the consumption of other addictive drugs. Moreover, there is a need for additional studies that identify patients or sub-types of patients with various addictions who might benefit from treatment with such medications: for instance, the possibility that polymorphisms in the *GLP-1R* genes influence the treatment outcome of GLP-1R agonists on alcohol drinking in patients with AUD. The preclinical findings that there is no tolerance development toward the GLP-1R agonists tested and that the decline in drinking persists after treatment discontinuation suggest clinical applicability (Suchankova et al., 2015; Vallof et al., 2015; Vallof et al., 2020). Although speculative and requiring confirmation in clinical trials, the GLP-1R agonists may have beneficial effects on both sexes, dose adjustments during long treatment periods might not be needed, and the effect may persist after treatment termination. Along with alcohol/drug addiction, individuals might develop addictions toward natural rewards, such as sex and palatable foods. As the GLP-1 pathways also modulate such responses (Vestlund and Jerlhag, 2020a; Vestlund and Jerlhag, 2020b), the possibility that patients with other addictions might benefit from GLP-1R therapies should be considered. Another patient group who might benefit from treatment with GLP-1R agonists is gastric by-pass patients who increase their alcohol intake after surgery. However, the efficacy of GLP-1R agonists to reduce drinking in this subgroup may diverge from those without surgery, as GLP-1 is increased after surgery (Gerner et al., 2014).

In conclusion, these preclinical and clinical studies point toward the possibility of using GLP-1R agonists to treat patients with addictions, an interaction that should be elucidated in future studies.
